# Advancements in Nanoemulsion-Based Drug Delivery Across Different Administration Routes

**DOI:** 10.3390/pharmaceutics17030337

**Published:** 2025-03-05

**Authors:** Maria D. Chatzidaki, Evgenia Mitsou

**Affiliations:** 1 Institute of Chemical Biology, National Hellenic Research Foundation, 11635 Athens, Greece; 2 Institute for Bio-Innovation, Biomedical Sciences Research Center “Alexander Fleming”, 16672 Vari, Greece; 3 Department of Molecular Chemistry and Materials Science, Weizmann Institute of Science, 6100 Rehovot, Israel

**Keywords:** nanoemulsions, drug delivery, (trans)dermal, oral, ocular, nasal, intra-articular

## Abstract

Nanoemulsions (NEs) have emerged as effective drug delivery systems over the past few decades due to their multifaceted nature, offering advantages such as enhanced bioavailability, protection of encapsulated compounds, and low toxicity. In the present review, we focus on advancements in drug delivery over the last five years across (trans)dermal, oral, ocular, nasal, and intra-articular administration routes using NEs. Rational selection of components, surface functionalization, incorporation of permeation enhancers, and functionalization with targeting moieties are explored for each route discussed. Additionally, apart from NEs, we explore NE-based drug delivery systems (e.g., NE-based gels) while highlighting emerging approaches such as vaccination and theranostic applications. The growing interest in NEs for drug delivery purposes is reflected in clinical trials, which are also discussed. By summarizing the latest advances, exploring new strategies, and identifying critical challenges, this review focuses on developments for efficient NE-based therapeutic approaches.

## 1. Introduction

Nanoemulsions (NEs) are nanometer-sized colloidal dispersions, typically consisting of an oil phase dispersed in an aqueous phase (oil-in-water, O/W) or vice versa (water-in-oil, W/O) with bicontinuous and multiple NEs (MNEs) (e.g., oil-in-water-in-oil (O/W/O) or water-in-oil-in-water (W/O/W)) representing additional categories [1,2]. Since the two phases are immiscible, they are stabilized by surfactants, with the final formulations exhibiting droplet sizes ranging from 50 to 500 nm [3]. These formulations have drawn significant attention due to their useful properties, with applications spanning pharmaceutics, cosmetics, the food sector, and other industries [4].

Among the different types of NEs, O/W NEs are widely used in pharmaceutical applications (e.g., Diprivan^®^ [5], Restasis^®^ [6]) as their external aqueous phase allows dilution in biological media. W/O NEs are less common but remain valuable, especially in dermal applications [7] and in vaccine development where they have been studied as adjuvants to enhance immune responses [8]. In addition, MNEs offer a platform for controlled release and combinational therapy. For example, W/O/W NEs have been investigated for oral insulin delivery, protecting the protein from gastric degradation, and enhancing its intestinal absorption [9]. In another case, they have been explored for the co-delivery of chemotherapeutic and imaging agents for theranostic applications [10].

The development of NEs is not as challenging and complicated as that of other nanomaterials; however, it requires a rational choice of compounds which not only affect the stability and the system’s ability to incorporate compounds but also strongly alter its final pharmacological effect and safety profile. As NEs are characterized by kinetic stability and thermodynamic instability, their development necessitates the input of external energy. For NEs’ development, the preparation procedures commonly involve high-energy methods [11] such as high-pressure homogenization, ultrasonication, and microfluidization, which are commonly employed to apply mechanical forces that split the preformed droplets into smaller nanodroplets. Alternatively, low-energy methods (phase inversion temperature or composition method) [12] have been explored, leveraging phase transitions induced by changes in temperature or composition. Nonetheless, high-energy methods remain the most frequently used due to their efficiency and ability to produce NEs with narrow size distribution and desirable physicochemical properties. Since structural properties, preparation methods, and physicochemical characterization have been discussed extensively elsewhere [13,14], this review will focus on recent advancements aimed at enhancing the efficiency of drug delivery via NEs.

The structure of NEs provides valuable advantages in drug delivery by increasing the solubility of the encapsulated compounds, protecting them from external degradation, and enabling them to cross biological barriers. This makes NEs highly effective in improving or altering the pharmacokinetic and pharmacodynamic profiles of therapeutic agents [15,16] with proven efficacy across various applications. In addition, NEs facilitate controlled and sustained drug release, reducing toxicity and minimizing off-site adverse effects due to their ability to be modified with targeting moieties. However, the application of NEs for delivering compounds of pharmacological interest faces various challenges. Their thermodynamic instability, along with the complex biological environment into which they are introduced (which contains proteins, enzymes etc.) can lead to destabilization and altered physicochemical properties, potentially impacting drug encapsulation and overall efficiency [17]. In parallel, the addition of stabilizing agents, alongside other components, may trigger unfavorable immune responses [18], ranging from mild to severe reactions (e.g., accelerated blood clearance (ABC) phenomenon [19] and complement activation-related pseudoallergy (CARPA) [20]). Furthermore, potential toxic effects, particularly those associated with surfactants—mainly in repeated dosing regimens—must be carefully evaluated [21]. Another significant challenge for NEs lies in the body’s clearance mechanisms. NEs, following administration, are often taken up by the cells (i.e., macrophages) of the reticuloendothelial system (RES) and eliminated through organs like the liver and kidneys, leading to rapid clearance from circulation [22]. This reduction in bioavailability makes efficient targeting essential to ensure that NEs reach the site of action before elimination. Apart from the biological standpoint and from a production perspective, the development and scalability of NEs also present considerable hurdles. Achieving consistent large-scale production of NEs with uniform size and reproducible properties is a complex and demanding procedure, while the lack of long-term stability can limit their ability to retain drugs over prolonged periods. 

Even with these challenges, the global NEs market, valued at USD 6858.26 million in 2023, is projected to grow to USD 9961.59 million by 2032 [23]. This growth is driven by the increasing demand for advanced drug delivery systems, cosmetics, and food products that offer improved drug solubility and enhanced stability. The pharmaceutical industry is a key driver of the NEs market [24]. North America and Europe currently dominate the market due to significant R&D investments and well-established pharmaceutical and cosmetic industries. However, the Asia–Pacific region is projected to experience the fastest growth, driven by the demand for innovative drug delivery systems, particularly in China, India, and Japan [25,26]. 

The market success of NEs (Table 1) and their numerous ongoing clinical trials (Table 2), which are anticipated to further propel market growth, are the result of NEs’ ability to efficiently deliver their cargo across different biological barriers in the human body. Pharmaceutical systems, whether NEs, polymeric nanoparticles or other formulations, must be capable of penetrating specific biological barriers and delivering their compound to the site of action at therapeutic relevant concentrations without off-site effects. The small size and tunable nature of NEs allow for improved penetration, controlled release, and enhanced bioavailability, making them ideal carriers for challenging-to-deliver drugs and thus expanding the treatment options for a variety of conditions while reducing the dose requirements and treatment-related cost.

## 2. Overview of NEs in Drug Delivery and Lessons Learned

The effectiveness of a drug or a drug delivery system relies on its ability to reach the target site and exert the intended effect. This “journey” includes concerns about chemical and physical stability [27], solubility [28], biomolecule interactions [29], targeting specificity [30], immunogenicity [31], and the need to overcome specific biological barriers depending on the administration route. These barriers serve as the body’s defense against antigens, including pathogens. Skin and mucosal membranes (such as those in the ocular and nasal cavities), as well as cellular and subcellular membranes, provide protection while also posing significant challenges for drug delivery. For example, the mucus in the nasal mucosa exhibits viscoelastic and adhesive properties that trap compounds while maintaining the necessary lubrication in the area [32]. Skin presents additional challenges due to its multiple layers that regulate permeability, while the blood–brain barrier (BBB) tightly controls molecular entry into the central nervous system (CNS) [33], preventing potential neurotoxic damage. Nanotechnology has been a tool since the late 20th century [34], for the efficient delivery of therapeutic components, facilitating higher solubility, protection, and controlled release of the encapsulated compound at the target site. NEs stand out among nanodelivery systems due to their ability to encapsulate a wide range of drugs, sustain release kinetics, and exhibit tunable physicochemical properties such as size, charge, and viscosity [35]. They also allow for surface modifications that enhance targeting [36] and prolong retention [37] at the site of action, while remaining relatively simple to develop and, in most cases, cost-effective. These properties contribute to improved permeability across biological barriers, enhanced bioavailability, and reduced drug toxicity [38]. In the evolving field of drug delivery (e.g., nucleic acid [39] and phototherapy-based treatments [40]), ongoing research on the development of NEs continues to address critical challenges, including tailored strategies such as permeation enhancers [41], mucoadhesive components [42], and ligand-based targeting approaches (e.g., cell-penetrating peptides [43] or receptor-specific compounds [44]) to optimize drug transport across specific barriers [45,46,47,48,49].

Among the administration routes, parenteral drug delivery has been extensively studied since the introduction of NEs in clinical practice, with well-established advantages [50]. Despite its effectiveness, parenteral delivery has limitations, including patient discomfort (especially in chronic and pediatric conditions), the need for trained personnel for administration, and an increased risk of infections (particularly in economically disadvantaged regions) [48]. These drawbacks have increased interest in alternative delivery routes, creating new opportunities for societal and economic impact. Many of the principles established in this field, however, proved beneficial in other administration routes. Properties such as particle size, surface charge, and biocompatibility significantly impact NEs’ performance across all routes. In parenteral applications, particle sizes are optimized (up to 200 nm) to avoid rapid clearance by the mononuclear phagocyte system (MPS) [51], knowledge that is also relevant for other routes. Functionalization with polymers such as PEG [52] has been adapted parenterally after the success of the liposomal Doxil^®^ [53]. Biocompatibility remains a key consideration across all routes, as excipients ranging from oils to surfactants have been shown to be safe first in parenteral applications, including castor, soybean, safflower oil, and medium-chain triglycerides (MCT) [54], Tweens^®^ [55], glycerin, poloxamers [56], and PEGs. Similarly, the extensive studies on selecting suitable surfactants and co-surfactants to ensure the stability of NEs are directly applicable to the formulation of NEs for oral administration, where they must withstand the harsh conditions of the gastrointestinal (GI) tract and the challenging mucus environments. Co-encapsulation strategies [57] along with biomimetic NEs [58] (i.e., NEs incorporating biological membrane fragments—such as red blood cells or immune cell-derived coatings—to enhance immune evasion and targeting efficiency following) have also been explored. The latter was first used in 2018 in an intraperitoneal injection of RBC-coated NEs in a hemorrhagic shock model for oxygen delivery, paving the way for their application in the routes discussed in this review.

Building on these foundational principles and on already-tested compounds for parenteral administration, NEs are further optimized and designed for other routes, each presenting unique biological barriers and challenges that require tailored formulation strategies.

## 3. Recent Approaches in NE-Based Drug Delivery

Building upon established principles and leveraging previously tested compounds and techniques, NEs continue to be optimized for many administration routes. Each route presents distinct biological obstacles—such as enzymatic degradation in the GI tract, limited mucosal permeability in nasal delivery, or the stratum corneum (SC) in transdermal applications—necessitating tailored formulation strategies. By fine-tuning properties such as particle size, charge, and surface functionalization, researchers are enhancing the stability, bioavailability, and therapeutic efficacy of NEs across different routes.

**Figure 1:**
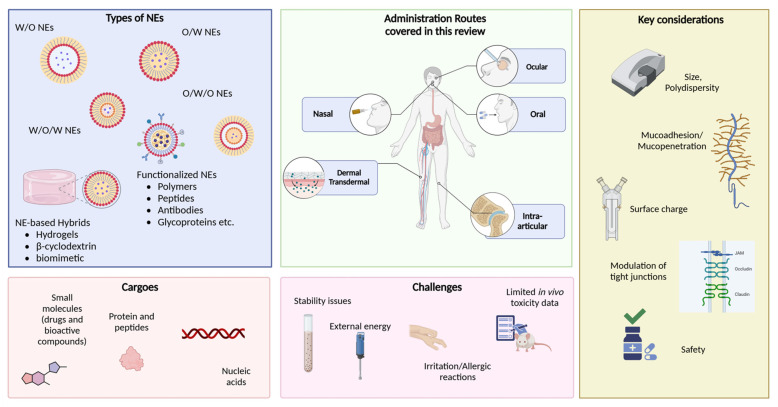
Graphical summary of NEs in drug delivery, illustrating different NE types, encapsulated cargoes, administration routes, and key challenges and considerations for effective formulation and application, as covered in this review. Created in BioRender. Mitsou, E. (2025) https://BioRender.com/v69p693, accessed on 1 January 2025.

In the present review, we discuss recent advancements and innovative approaches to overcome the challenges faced by NEs in drug delivery, broadening the scope of their clinical applications the last five years (2019-to-2024) through the administration routes currently in the spotlight: (trans)dermal, oral, nasal, ocular, and intra-articular routes (Figure 1). Our search strategy included an initial query in (a) Google Scholar and (b) PubMed search engines using the keywords “specific route of administration (e.g., nasal, intra-articular, etc.)” + “nanoemulsions”, with relevant filters applied for publication dates. In the second step, the results were carefully reviewed for relevance to the focus of this review, specifically targeting studies involving NEs with mucoadhesive compounds, peptides, and other targeting moieties, or those related to vaccines and photothermal/photoacoustic therapy due to the growing interest. Many studies were excluded at this stage, primarily due to their lack of novelty or absence of in vitro/in vivo supporting data. Earlier studies (prior to 2019) are also cited where necessary to provide sufficient background.

### 3.1. (Trans)Dermal Administration

Drug transport across the skin involves two main pathways, with the SC serving as the primary barrier. This thickest layer of the skin acts as a major barrier for drug molecules. There are two main routes for drug penetration: the intercellular lipid route and the transcellular route. In the intercellular lipid route, bioactive molecules travel between skin cells, using the spaces between corneocytes [59]. Lipophilic or amphiphilic molecules can interact with the lipophilic regions of the SC, filling gaps between cells and lipid layers to aid in transepidermal penetration. Hydrophilic molecules mainly diffuse laterally through areas with less water. In the transcellular route, molecules pass directly through skin cells via passive diffusion deep into keratin, contributing to the SC’s integrity. This type of diffusion is commonly used in dermal applications for cosmetic purposes and local disease treatments and should not be confused with transdermal drug delivery (TDD) [60], which is mainly used for compounds to reach the bloodstream, avoiding the first-pass effect. Follicular delivery [61] is also a potential delivery option, but in vitro study limitations and inconsistencies in mouse models highlight the need for more reliable evaluations and are not further discussed in the present review [62].

The goal of dermal applications, as previously mentioned, is the topical delivery of drugs or bioactive compounds. Recent advancements in topical delivery for skin cancer treatment have used NEs, emerging as a promising strategy to enhance therapeutic efficacy while minimizing side effects. NEs improve skin permeation and retention of therapeutic agents, enabling localized treatment directly at the tumor site. The oils and surfactants selected for these specific applications are carefully chosen to be compatible with skin tissues, minimizing irritation and reducing the risk of adverse reactions [63,64]. A recent study developed NEs for the topical delivery of piplartine, a cytotoxic agent. The piplartine-loaded NEs were modified with chitosan and sodium alginate and evaluated for their ability to deliver the drug and their therapeutic efficacy. The cytotoxicity assays showed that piplartine’s effectiveness was approximately 2.8 times higher when delivered via chitosan-modified NEs compared to its solution, with an IC50 value of 14.6 μM against melanoma cells [65]. NEs and gels have been proposed as carriers for daidzein, a lipophilic isoflavone with potential health benefits including anti-inflammatory and anticancer properties. The cytotoxicity assays indicated that the NE-based formulations enhanced the anticancer activity of daidzein, demonstrating a higher efficacy in inhibiting cancer cell growth than non-NE forms [66]. In addition to NE-based gels, decorated MNEs have emerged as promising alternatives for topical skin applications. Specifically, chitosan-decorated MNEs have shown potential in improving the delivery of therapeutic agents through the skin by enhancing drug permeation and retention. This MNE was developed using a two-step emulsification process, with chitosan incorporated as a coating agent to facilitate deeper skin penetration and controlled drug release. Ex vivo permeation studies revealed that chitosan decoration significantly enhanced drug permeation through rat skin compared to non-decorated formulations and improved skin retention capabilities [63]. Psoriasis, a chronic inflammatory skin disorder, has also been targeted for treatment with drug-loaded NEs designed for topical application. Researchers developed a curcumin-loaded NE for psoriasis treatment, incorporated into a polymeric hydrogel matrix. In vitro studies revealed that the hydrogel significantly improved curcumin skin permeation compared to conventional formulations, highlighting the formulation’s potential for topical use [67].

Scholars in the cosmeceutical field are also actively exploring advanced technologies to enhance the delivery of bioactive compounds, leading to innovative products. Utilizing saponins in NEs has recently been proposed to increase the solubility and bioavailability of α-tocopherol. Release studies have demonstrated a controlled release profile, indicating that this NE system can effectively deliver α-tocopherol to the skin [68]. Another study aimed to evaluate the clinical efficacy of NEs containing *Macadamia integrifolia* seed oil for enhancing skin hydration and reducing the appearance of wrinkles, compared to conventional emulsions using Tween^®^ 80 and Span^®^ 80 as surfactants. Both types of formulations significantly improved skin hydration and reduced wrinkles in participants. However, the NEs outperformed the conventional emulsions in enhancing skin hydration levels [69]. By leveraging the unique properties of NEs, researchers are developing innovative cancer-preventive skincare products. For instance, NEs have shown great promise in enhancing the effectiveness of sun protection formulations. Their structure allows for the efficient encapsulation and delivery of active ingredients, leading to improved UV protection and enhanced skin penetration [70]. A recent study focused on developing NEs loaded with natural antioxidants from olive mill wastewater and phycocyanin to offer both effective UV protection and beneficial antioxidant effects, promoting healthier skin. The results showed that the antioxidant-loaded NEs had significant UV absorption capabilities, indicating their potential effectiveness in shielding the skin from harmful UV radiation [68]. Additionally, ascorbic acid, commonly known as vitamin C, is a powerful antioxidant with notable skincare benefits. To improve its penetration abilities and address its poor solubility, researchers encapsulated it in NE patches. These patches were created by incorporating 80 mg/mL of ascorbic acid into an O/W NE, which was then blended with a gel-forming solution to produce a stable patch suitable for dermal applications. The findings suggested that NE-based patches could be a promising method for delivering ascorbic acid to the skin, enhancing its stability and bioavailability [71].

**Figure 2:**
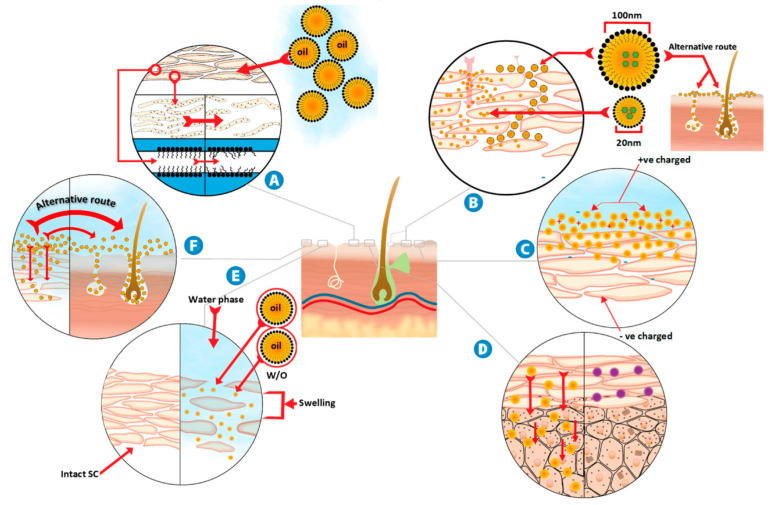
Mechanisms of transdermal enhancement of hydrophobic drugs from NE: (**A**) Disruption of lipid bilayer of the stratum corneum (SC). (**B**) Enhancement of transdermal permeation through oil droplet nano-sizing. (**C**) Binding of positively charged NE to negatively charged skin. (**D**) Changing drug partition into skin layers. (**E**) Hydrating skin and the dilation of the SC intercellular channels. (**F**) Changing the permeation pathway of lipophilic permeants to follicular delivery with an O/W NE. Obtained under the terms of the Creative Commons CC BY license from [54].

NEs have emerged as a promising technology also in TDD due to their unique properties, including small droplet size, high surface area, and the ability to encapsulate both hydrophilic and hydrophobic drugs with improved abilities compared to conventional emulsions or microemulsions. More specifically, NEs offer significant advantages over traditional emulsions, including lower viscosity and a larger interfacial area, which enhance the solubility and bioavailability of poorly water-soluble drugs. In terms of stability, NEs demonstrate high kinetic stability and resist gravitational separation due to their small droplet size. This helps prevent droplet flocculation, ensuring that the system remains stable during storage for a longer period compared to conventional emulsions. NEs can be formulated into a variety of dosage forms, such as gels, creams, foams, aerosols, and sprays, and can be administered through multiple routes, including oral, topical, intravenous, and intranasal. Compared to microemulsions [72], NEs require lower concentrations of emulsifiers, resulting in less toxic formulations. These features make them ideal for enhancing the bioavailability of various therapeutic agents. A recent review on NEs for transdermal applications highlights components like oleic acid, Capryol^®^ 90, and isopropyl myristate (IPM) for their effective skin permeation capabilities used as the NEs’ oil phase. Additionally, the inclusion of alcohols or short-chain polyols can increase the efficiency of NEs in penetrating the SC. Additionally, common penetration enhancers (accelerants) used in these formulations include fatty acids, esters like ethyl oleate, terpenes such as limonene, menthol, and cineole, as well as other known ingredients like Azone^®^ and dimethyl sulfoxide (DMSO). These molecules are effective because they interact with the SC’s lipid layer, improving the overall system’s penetration ability while maintaining low toxicity levels [62] (Figure 2). A recent study focused on developing and optimizing a NE for insulin delivery. Addressing the shortcomings of traditional insulin administration methods, the study indicated that the new system significantly enhanced drug’s permeation compared to conventional formulations, demonstrating its potential as an effective TDD system. In vitro findings were corroborated by in vivo evaluations, which showed promising results regarding insulin release and action duration [73]. In another study, researchers developed sulconazole-loaded NEs by screening different mixtures of oils such as MCT or olive oil and mixtures of surfactants and co-surfactants (Tween^®^ 80 or Labrasol^®^) to optimize the drug’s solubility. It was found that the carrier enhanced the drug’s permeation through the skin, and it improved its antifungal activity against dermatophytes [74]. For transdermal applications, advanced systems like NEs gels have been proposed as superior candidates for drug penetration, building upon the classic NE technology. Specifically, a recent study explored the formulation and evaluation of NEs containing asiaticoside (ASI), a compound known for its therapeutic effects on hypertrophic scars but challenged by its large molecular weight, low water solubility, and poor lipophilicity. ASI-loaded NEs and NEs-based gels were developed to enhance the transdermal delivery of ASI. The ex vivo studies confirmed that both formulations effectively overcame skin barrier properties and ensured safety, establishing them as promising candidates for the transdermal delivery of ASI [75]. The formulation and effectiveness of a cationic NE gel to enhance the transdermal delivery of rifampicin, a crucial antibiotic for treating tuberculosis, was also recently explored. Using Design Expert^®^ software (version 8.0.7.1, Stat-Ease, Inc., Minneapolis, MN, USA), researchers optimized the formulation parameters, focusing on the surfactant ratio of Capmul^®^ and Labrasol^®^. In vivo studies revealed that the maximum concentration and area under the curve for transdermal application were 4.34 and 4.74 times higher, respectively, than those achieved through oral administration while reducing adverse effects associated with conventional methodologies. This research underscores the potential of advanced NE technology to improve drug delivery systems for managing tuberculosis infections effectively [76].

Overall, modifying physicochemical properties of drugs improves the penetrability through the SC or epidermal layer and physical interaction with cellular membranes. Factors such as smaller droplet size, high zeta potential, low polydispersity index, high elasticity, and the choice of NE type and emulsifiers play crucial roles in this process. Surfactants alter the cellular integrity of the skin in several ways through which skin hydration takes place. In the case of human subjects, skin hydration time can be increased due to the presence of thicker SC layer. However, lipidic NEs with droplet size above 50 nm are less likely to penetrate through passive diffusion and require a concentration gradient or external assistance that may drag those droplets in to the deeper layer of the skin. For effective transdermal delivery, the size of the lipid NE droplets should ideally be below 50 nm as skin permeability is inversely related to droplet size. The charge of the skin surface affects dermal or transdermal delivery design. Negatively charged skin favors better interaction with the positively charged nanodroplets; however, negatively charged droplets show no/very little interaction with the skin cells if the NE is stabilized by surfactants. This highlights the importance of optimizing NEs’ properties for efficient drug delivery. 

### 3.2. Oral Administration 

The GI tract has been widely proposed as an effective administration site of bioactive compounds even though it presents significant challenges due to enzymatic degradation, acidic pH, and limited permeability of the intestinal wall. NEs have shown potential for improving nutrient absorption and enabling direct cellular uptake. However, their toxicity profile is a concern, influenced mainly by their surfactant content [77]. Reactive ingredients like β-carotene have been proposed to significantly increase the toxicity of NEs, potentially offsetting the benefits of enhanced nutrient delivery [78]. Recent advancements in the formulation of NEs for oral administration have highlighted their potential in enhancing drug delivery, particularly for poorly soluble drugs and therapeutic agents. A recent study found that the composition of NEs could either enhance or inhibit the absorption of bioactive compounds. This suggests that the design of the carrier of bioactive molecules is critical in overcoming biological barriers [79]. The use of NEs enhances the intestinal absorption of benzopyran HP1, a compound with significant analgesic properties but limited by poor water solubility, which restricts its oral bioavailability. The research employed the Ussing chamber model and rat jejunum to examine the transport mechanisms involved. The findings revealed that the apparent permeability coefficient of benzopyran HP1 increased by approximately 5.3 times when delivered via NEs compared to its free form. Also, an ex vivo mucoadhesion test revealed that 50% of NEs particles adhere to the intestinal mucus in 30 min, contributing to the enhanced absorption before the mucus turnover timeframe (50–270 min). Additionally, the particle size (~150 nm) was suitable for endocytosis-mediated transport, further supporting effective delivery mechanisms [80]. The selection of emulsifiers plays a crucial role in the oral administration of bioactives. A recent study investigated how different emulsifiers impact the bioaccessibility and bioavailability of β-carotene when delivered through NEs. The study found that NEs stabilized with lecithin had significantly higher levels of β-carotene in the GI-digested micellar fraction compared to those stabilized with sodium caseinate, indicating that lecithin probably facilitates the bioactive’s bioaccessibility. Despite lower amounts of β-carotene in the apical compartment, lecithin-stabilized NEs led to higher cellular content within Caco-2 cells, demonstrating improved absorption efficiency while both emulsifiers triggered an inflammatory response [81].

Natural polymers like alginate and aloe vera for the formation of NEs, have also been proposed as good candidates for oral delivery. More specifically, W/O/W MNEs coated with alginate and aloe vera gel, created through an ionic gelation process using calcium chloride, have been proposed for oral insulin delivery. The results demonstrated that these formulations maintained their structural integrity, effectively protecting insulin from stomach degradation. Additionally, studies using Caco-2 cell lines showed a 20% to 25% increase in insulin translocation across the cell monolayer, indicating that the proposed systems could enhance insulin absorption into the bloodstream [82]. A recent study explored a NE formulation to enhance the oral bioavailability of ibuprofen, a widely used nonsteroidal anti-inflammatory drug with poor solubility and GI absorption. Researchers utilized a ternary phase diagram to optimize the concentrations of olive oil (the oil phase), sucrose esters (the surfactant), and glycerol (the co-surfactant). In vitro studies showed that this ibuprofen-loaded NE significantly improved drug absorption compared to conventional formulations. Finally, an in vivo study in rats revealed that the NE’s oral bioavailability was approximately twice that of an oil solution, indicating its potential as an effective delivery system for ibuprofen [83].

**Figure 3:**
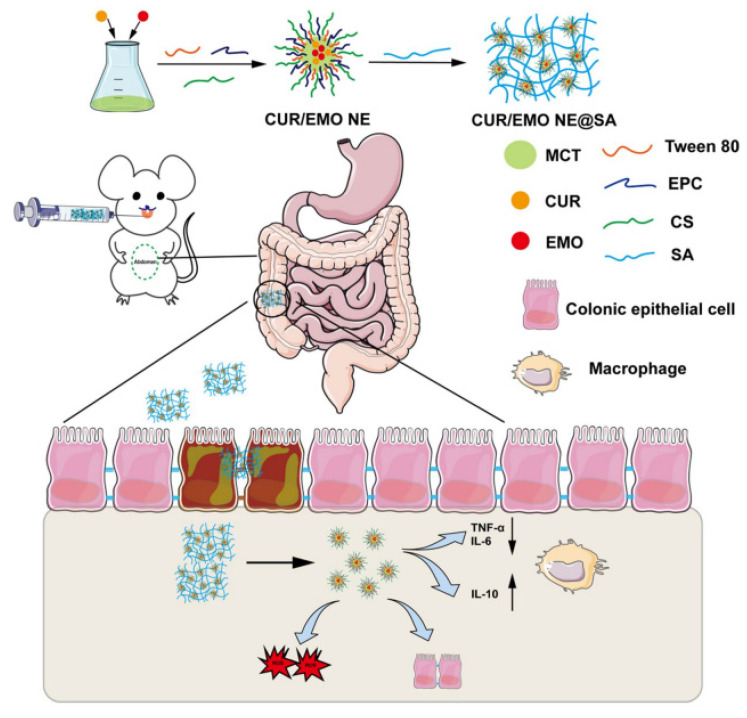
Schematic of preparing curcumin/emodin (CUR/EMO) NE by high-energy emulsification and using the physical cross-linking of chitosan and sodium alginate (SA) for CUR/EMO NE@SA, as well as demonstrating the advantages of CUR/EMO NE@SA in alleviating inflammatory bowel disease (IBD). Obtained under the terms of the Creative Commons CC BY license from [86].

The therapeutic potential of NEs in protecting against GI damage has also been investigated, demonstrating the benefits of encapsulating bioactive compounds in these matrices. One study highlighted the protective effects of a curcumin-loaded NE against indomethacin-induced intestinal damage. In animal models, this NE significantly reduced intestinal injury and inflammation compared to untreated controls, with histopathological examinations showing the protection of the intestinal mucosa [84]. Similarly, researchers examined the protective effects of a silymarin NE against GI toxicity caused by 5-fluorouracil (5-FU), a chemotherapeutic agent known for its severe GI side effects. The silymarin NE significantly mitigated 5-FU-induced GI toxicity in rats, resulting in improved intestinal morphology and lower inflammatory markers compared to control groups [85]. Another study introduces an innovative oral hydrogel NE designed for the co-delivery of anti-inflammatory agents to treat inflammatory bowel disease (IBD). This formulation aimed to enhance the therapeutic effects of its components while promoting the repair of intestinal mucosa. In animal models of IBD, the hydrogel NE effectively reduced inflammation and supported healing [86] (Figure 3). These findings indicate that NEs could be developed into effective therapeutic options for GI disorders associated with various pharmacological treatments, including nonsteroidal anti-inflammatory drugs (NSAIDs), chemotherapy, and IBD. In a different context, studies by the same team highlight the effectiveness of essential oil-encapsulated NEs, specifically containing thymol and eugenol, as dietary supplements for broiler chickens to improve growth performance and GI health. These formulations resulted in significant increases in body weight gain and improved feed conversion ratios. Additionally, they promoted beneficial gut microbiota and upregulated genes associated with digestive enzymes and intestinal barrier functions. The thymol NE provided protection against *Salmonella typhimurium*, while the eugenol NE was effective against *Escherichia coli* O78, demonstrating their potential in safeguarding against specific bacterial infections [87,88].

Studies from the last five years concerning NEs for oral administration suggest that they significantly enhance the bioavailability, stability, and absorption of poorly soluble drugs and bioactive compounds. Research highlights their role in improving intestinal permeability, protecting drugs from enzymatic degradation, and facilitating targeted delivery. Innovations such as polymer-coated NEs, hydrogel formulations, and optimized emulsifiers have further improved drug transport and therapeutic efficacy. Additionally, NEs have demonstrated protective effects against GI toxicity and inflammation, with potential applications in treating conditions like IBD and enhancing gut health. These findings underscore the growing relevance of NEs in advancing oral drug delivery systems.

### 3.3. Ocular Administration

The human eye is a challenging target for drug administration (whether to its anterior or posterior part) due to the complex ocular barriers present, including (a) the tear film (pre-corneal site), (b) the cornea, (c) the conjunctiva, (d) the sclera and choroid (e), the blood–ocular and (f) the blood–retina barriers, often necessitating frequent dosing or invasive techniques [89]. Despite its challenging structure, ocular drug delivery is of outmost importance, considering that >480 million people suffer from partial or total vision loss attributed to diseases or aging (e.g., glaucoma, age-related macular degeneration) [90]. Dry eye disease and fungal keratitis are some of the clinical manifestations that have gained significant attention, with numerous formulations—including ointments [91], gels [92], and contact lenses [93]—being developed for treatment purposes. Apart from the challenging physiological anatomy of the eye, lacrimal drainage, pre-corneal loss and the trans-conjunctival systemic absorption result in only 5% of topically applied drugs reaching their target [94], while orally and intravenously administered doses are mainly hindered by the low blood–ocular flow.

NEs have been employed in ocular drug delivery, with the first FDA-approved product (Restasis^®^, cyclosporine NE for dry eye disease) receiving approval in 2003 (application number 021023) [95]. They offer increased retention time and effective penetration across the corneal tight junctions compared to conventional non-nanoparticulate formulations, while their viscosity can be modulated in such a way to increase their retention time and the pharmacological effect further [96]. For NEs to efficiently resist rapid clearance from the lacrimal fluid [97] and enhance permeation through the multi-layered corneal barrier (see review [98]) specific physicochemical properties should be fulfilled to minimize aggregation and potential visual disturbances among others. Nanodispersions with particle sizes of below 200 nm are preferred, while the refractive index of the system (a marginalized property in other drug applications) should remain between 1.34 and 1.36 to avoid blurring, with pH values within the range of 6.6 to 7.8 to prevent chemical injury to the eye.

The selection of ophthalmic NEs’ components, apart from their pharmaceutical acceptability must be made carefully. NEs’ components first permit the interaction with the lipid tear film in the eye (three-layer film responsible for light refraction, lubrication, defense from antigens, and transport of metabolic compounds) [99], increasing the retention time even further. Surfactants are responsible for reducing the contact angle between the eye drop and the cornea [96], while enhanced ocular retention time can be achieved, such as in the case of tacrolimus loaded cationic NE in the presence of the cationic cetalkonium chloride [100], by electrostatically interacting with the mucin layer of the tear film. Cationic surfactants have been implemented in different compositions for the delivery of corticosteroids such as triamcinolone acetonide—a synthetic glucocorticoid with anti-inflammatory and immunomodulatory properties—to provide safest alternative to the intravitreal injections by topically applied NEs. O/W soybean oil/Tween^®^ 80/poloxamer188/1,4-diazabicyclo[2.2.2]octane derivative (DABCO, cationic surfactant)/glycerol NEs have been developed. DABCO is a cationic quaternary ammonium found in natural physiologically active compounds with antibacterial activities. The system proved to be non-toxic first in vitro and subsequently in vivo, while the inflammation of the anterior segment was statistically significant from the free drug attributed to its sustained release and spreadability [101,102]. However, the presence of surfactants may cause irritation, and cataractogenic effects have even been reported [103], while increased viscosity could lead to blurred vision and discomfort for the patient, underlining the importance of careful adjustment. Surface located components that increase the NEs’ surface charge (either surfactants or other additives such as stearylamine) can lead to increased electrostatic interactions with the anionic mucins, increasing the residence time shortly after application. For example, cetyltrimethylammonium bromide (CTAB) is a cationic surfactant with mucoadhesive properties that has raised concerns regarding its biocompatibility in many applications; however, different in vivo tests (e.g., ocular hypotensive effect test and Draize ocular irritation test) proved its non-toxic nature in the eyes of rabbits [104].

Oil phase of NEs is generally including compounds such as IPM [105,106], Capmul^®^ MCM [107], Mygliol^®^ [108,109], etc., while there is a trend (as in the previous cases) towards natural oils. Olive oil [110], turmeric oil [111], or cannabidiol [112] have been implemented in NE structures because of their ability to solubilize highly hydrophobic drugs along with additional antibacterial/inflammatory/oxidant properties without compromising the biocompatibility of the systems. In vivo studies have proved that incorporation of 0.4 to 1.6% CBD *w*/*v* in NEs dispersed phase reduced the levels of key inflammatory cytokines while reducing intraocular pressure in murine models [112].

Mucoadhesive properties are crucial for ocular delivery, particularly with pH-, ion-, and thermo-responsive compounds playing a central role in the in situ formation of gels, for interaction with the biological barrier. Chitosan as a cationic compound (pH responsive) has also been implemented in dry eye disease to protect or supplement the tear film, offering improved retention time and lubrication. An O/W system of Mygliol^®^ 812/chitosan/lecithin/Kolliphor^®^ EL developed through microfluidization process. In this case, chitosan was incorporated into the system through the interaction with lecithin covering the nanodroplets’ surface while offering additional antibacterial properties. The system showed increased mucoadhesion in in vitro tests; however, no further in vivo experiments were conducted [108]. Chitosan has also been implemented in NEs for the treatment of ocular tuberculosis [113]. Ion-sensitive in situ NEs have been used to increase retention time, as in the case of glaucoma, where brinzolamide has been incorporated into a castor oil/polyoxyl-35 castor oil/Tween^®^ 80 NE. The systems had a size smaller than 200 nm, and an anionic heteropolysaccharide, gellan gum (0.3–1% *w*/*v*), was used as the in situ gelling agent, with a gelling time of less than 15 s. Accelerated conditions tests lead to stable systems regarding their physicochemical properties while in vivo toxicity studies importantly revealed no histopathological findings in rats and rabbits [114,115]. Thermosensitive poloxamers such as poloxamer 188 [116] and 407 [117,118] have been introduced in NEs to increase retention time with their mucoadhesive properties, and it has been reported that these can temporarily open tight junctions between the epithelial cells [119].

**Figure 4:**
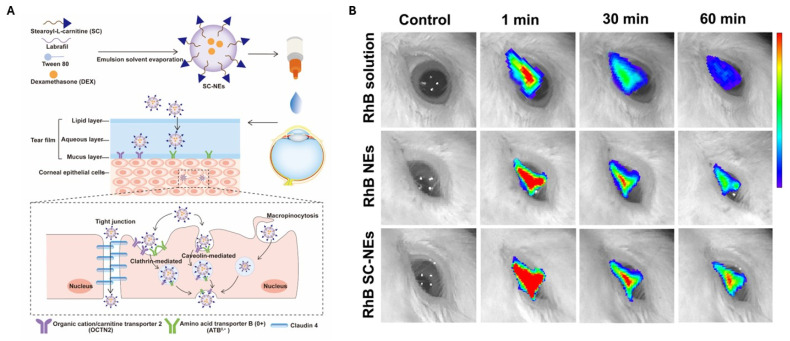
(**A**) Schematic representation of a stearoyl L-carnitine-modified NEs (SC-NEs) for the ocular delivery of dexamethasone along with the proposed transport mechanisms for SC-NEs in human corneal epithelial cells (HCECs). SC-NEs and Na^+^/Cl^-^ bind to the specific sites of OCTN2 and ATB^0,+^, and the transporters change their conformation from outward-facing to a stable occluded state, triggering membrane invagination and clathrin- and caveolin-medicated endocytosis process. In addition, macropinocytosis and caveolin and clathrin-independent pathway are also involved in the cellular uptake and transport of SC-Nes. (**B**) Ocular surface retention study of SC-NEs in rabbit eyes. Fluorescence images of rabbit eyes after topical instillation of rhodamine B (RhB) solution, RhB NEs, and RhB 10% SC-NEs at different time points. Obtained under the terms of the Creative Commons CC BY license from [121].

However, surface charge changes are not a straightforward approach to specifically target cell populations. It is known that positively charged NEs electrostatically interact with the negatively charged glycosaminoglycans and hyaluronic acid, hindering their approach to the retina while increasing the formation of protein corona and decreasing cellular uptake. To address these challenges, a research group modified NEs for gene delivery with cleavable phosphate groups. Initially, the negative charge, through the incorporation of tripolyphosphate (z-potential: −22 mV), facilitates penetration through the negatively charged mucus, avoiding interactions. Subsequently, cleavage by alkaline phosphatase (ALP) in the retina changes the surface charge (z-potential: +10 mV; shifting in 2 h), promoting cellular internalization by exposing the poly-L-lysine. This ALP-mediated cleavage is particularly relevant as ALP is present in various retinal cell types, including Müller cells and cone photoreceptors. The resulting CPP-coated NE, formulated using IPM, Capmul^®^ MCM, Tween^®^ 80, and propylene glycol exhibited a particle size of 146 nm. Importantly, no in vitro toxicity was observed after 1–3 h of incubation with the 661 W cell line, although no in vivo results have been reported to validate this approach [120]. Other NEs have been modified with amphiphilic stearoyl L-carnitine to target the organic cation/carnitine transporter 2 (OCTN2) and amino acid transporter B for superior corneal permeation, ocular retention, and the in vivo anti-inflammatory activity of dexamethasone (DEX) [121]. Following in vivo administration, the corneas were removed, and it was found that 10% of the amino acid lead to stronger fluorescence intensity, indicating increased corneal permeation, while ex vivo results proved the transcorneal permeation of DEX by 1.2-fold compared to the non-carnitine NEs. One of the few ocular retention time studies demonstrated that the unmodified NE increased the retention of rhodamine B, while amino acid modification further enhanced it (Figure 4). Additionally, the system reduced the inflammatory response and downregulated multiple markers.

Interestingly, NEs have proven to be not only effective drug delivery systems but also efficient lubricants. This dual action is particularly advantageous in managing dry eye disease and has enabled their advancement into clinical trials (Table 2). Specifically, Systane^®^ Complete [122] lubricant eye drops (Alcon Laboratories, Inc., Fort Worth, TX, USA) represent an innovative propylene glycol-hydroxypropyl-guar (PG-HPG) NE designed to address deficiencies in both the lipid and aqueous layers of the tear film. HPG increases in viscosity when exposed to tear film (a three-orders-of-magnitude increase) due to cross-linked in situ borate ions gel. Lipids, particularly phospholipids—the most abundant lipids in tear film—are key to the superior lubrication of the eyes. The NEs’ droplets act as depots for the delivery of the anionic phospholipid dimyristoyl phosphatidyl glycerol. The product provides protection, improves cell barrier function, and enhances the lubricity [123] of the corneal epithelium, all of which are critical for the management of dry eye disease. In a phase 4, open-label clinical trial the product was self-administered by 183 patients with different forms, twice a day for 28 days. The treatment demonstrated a low incidence of adverse effects, with only 1.5% of participants withdrawn. The trial showed a significant decrease in discomfort scores by day 14 and a reduction in corneal staining scores by day 28 compared to baseline [122,124] (Table 2).

As in other applications, NEs have been combined with different systems to enhance the pharmacological effects of their encapsulated compounds. For example, Soe et al. [118] demonstrated that tacrolimus can be complexed with β-cyclodextrin to improve its aqueous solubility. This complex was subsequently incorporated into zein nanoparticle-stabilized NEs, resulting in increased entrapment efficiency and enhanced mucoadhesive properties compared to relevant controls. The formulation exhibited a mean particle size of 200 nm with narrow size distribution (PDI = 0.19 ± 0.02), producing a milky appearance and a negative zeta potential carrier (~−30 mV). It also showed low viscosity (3.38 cPs) and maintained extensive stability for up to six months. The system was non-toxic, non-irritant, and demonstrated high cellular uptake in anterior and posterior segmental cells in an in vitro Transwell dual-chamber model. The combination of the mucoadhesive properties of zein nanoparticles with the fluidity and spreadability of the NE appears to synergistically enhance the overall mucoadhesive properties of the system, highlighting the importance of these attributes.

NEs in ocular drug delivery have been studied extensively but a significant challenge lies in the limited use of well-characterized in vivo models. Most research has focused on irritation assays and in vitro/ex vivo permeability studies, with relatively few exploring [100,110,121] biodistribution in the eyes of animal models, likely due to the challenging handling requirements of ocular tissues. Furthermore, while the mucoadhesive properties of NEs are frequently highlighted, these claims are not supported by in vivo results. Scale-up and manufacturing also pose hurdles, as maintaining stability during large-scale production remains problematic. It is worth noting that while many systems demonstrate promising in vitro results, their translation to in vivo outcomes and eventual clinical success remains limited. Currently, two clinical trials are ongoing for Systane^®^ COMPLETE Lubricant Eye Drops. One (NCT06188260) is a Phase 2 study at the recruiting stage with participants aged 20–50 years with dry eye disease after 2 weeks of use. The other (NCT05724446) focuses on an O/W NE of clobetasol propionate, also in the recruiting stage of a Phase 3 (multicenter, randomize, evaluator-blinded) study for the treatment of inflammation and pain after cataract surgery in a pediatric population (0–3 years, 1 drop/day for 14 days). The systems have been successfully tested on adults in post-cataract surgery management with superior results over placebo in reducing inflammation (NCT04246801, NCT04249076) [125].

Overall, small particle size, viscosity modulation, refractive index, pH, and surface charge are physicochemical properties that need to be carefully optimized for effective ocular drug delivery, while mucoadhesive and gelling properties are also of great importance, always considering the specific needs of the eye. The small particle size (<200 nm) enhances corneal permeation, while the components of NEs contribute to the opening of tight junctions between epithelial cells, allowing access. A positive surface charge prolongs residence time in the mucus through electrostatic interactions, while rapid gelation helps resist lacrimal clearance. With the ocular products currently on the market and several ongoing clinical trials (Table 2), advancements in NE-based ocular systems could lead to many more products, with new directions emerging from novel formulations such as cleavable NEs, gene delivery systems, and lubricating formulations.

### 3.4. Nasal Administration

The nasal cavity is lined by the inner layer of nasal mucosa which serves as a protective layer of the respiratory tract while simultaneously facilitating gas exchange and drug absorption. The multi-cellular environment of epithelial, mucus-secreting goblet, and immune cells combined with a dense underlying vesicular network creates a dynamic and highly selective barrier which provides defense against inhaled antigens but also serves as an alternative, non-invasive route for drug delivery. The large surface area due to the microvilli presence, the high vascularization, and the relatively low enzymatic activity (compared to oral or other parenteral routes of administration) are the unique physiological features of the nasal mucosa favorable for attractive pharmaceutical applications [126]. The rich vascular network offers enhanced permeability and, most importantly, rapid absorption, which leads to quick systemic drug uptake [127], bypassing the first-pass metabolism while participating in the quick onset of action, dose reduction, and decreased side effects, especially in off-target organs. In parallel, nasal administration is convenient, non-invasive, and self-administered, which is attractive for chronic treatments or when administered to unconscious people (emergency cases) [128]. Apart from this, access to the brain by surpassing the BBB through the olfactory and trigeminal nerves has attracted significant attention from the scientific community [129]. However, the nasal mucosa also presents challenges, as its mucus layer traps foreign substances [130] and the rapid removal of formulations occurs through the mucociliary clearance process. In addition, low intrinsic permeability for hydrophilic and high-molecular-weight (HMW > 1 kDa) substances (e.g., peptides, proteins) [131], enzymatic degradation [132], and limited volume of administration (100–150 μL is commonly required) [133] are parameters that limit the use of this administration route.

NEs for nasal administration were first reported in the early 2000s [134], and research has intensified in the past decade. NEs have proven valuable for the localized treatment of conditions like nasal infections and inflammation (e.g., rhinitis, sinusitis, and allergic or infectious diseases) as they prolong drug retention in the nasal cavity [135]. Additionally, NEs can effectively target the brain through nose-to-brain delivery by utilizing the olfactory and trigeminal nerves, which provide a direct anatomical connection between the nasal cavity and the central nervous system (CNS) [136]. This method shows great potential for treating neurological disorders, such as Alzheimer’s disease [137], and administering psychiatric medications [138]. Moreover, intranasal vaccination has gained significant interest as a non-invasive immunization strategy [139].

The mechanism of drug transport in the nasal mucosa involves mainly two routes both applied in free- and nanodispersion encapsulated drugs: the transcellular route (through the cells) and the paracellular route (between cells, through tight junctions). Lipophilicity and molecular weight dictate the route of transport with lipophilic drugs crossing easily via the transcellular route, whereas hydrophilic drugs cannot [140]. Incorporating drugs into NEs helps them interact better with cell membranes (small droplet size and lipid content) or open tight junctions, allowing them to cross the epithelium. The modifiable nature of NEs allows them to adhere better to the mucosal surface and offer sustained drug release, increasing residence time in the nasal cavity compared to drug solutions, which are cleared quickly and require more frequent dosing. In addition, the NEs’ size plays a significant role as droplet size <200 nm is ideal for efficient absorption via transcellular channels and longer retention on the mucosa, whereas larger droplets (>200 nm) are more easily cleared. Studies have shown that formulations with diameters between 80 and 200 nm can remain on the nasal mucosa for up to 12–16 h, enhancing drug delivery, while larger droplets are cleared within 4 h [141].

Different drugs have been encapsulated in NEs for nasal delivery, such as clozapine [142], paclitaxel [143], and natural bioactive compounds, namely melatonin [144] and cannabidiol [145], either separately or through co-administration, as in the case of curcumin–quercetin [146,147]. Additionally, mRNA has been encapsulated in these systems for immunization against SARS-CoV-2 and others [148]. Interestingly, it is claimed that NEs can serve as an alternative to well-established lipid nanoparticles (LNPs) due to their composition, which is based on regulatory acceptable biomaterials and simplicity, allowing them to be easily translated to a global scale. The most used compounds for the formulation of NEs do not differ substantially from those used in other NE applications. Tweens^®^ (most representative Tween^®^ 80) is the main category of surfactants along with Transcutol^®^, Carbitol^TM^, Lauroglycol^TM^ 90, and Spans^TM^, which are non-ionic and of low toxicity. Oil phase mainly is composed of natural-based oils (almond, clove, peppermint, eucalyptus), vitamin E or Captex^®^ 800 and phospholipids, while polymers are used to increase mucoadhesion (chitosan, poloxamers) or stability and immune avoidance/recognition.

Efficient and targeted (intra)nasal drug delivery using NEs can be achieved through several key approaches, mainly involving (a) mucoadhesion [149] and/or mucopenetration [150] enhancement, which are critical for prolonging the residence time of NEs and penetrating the mucus barrier, respectively; (b) receptor-mediated targeting [151], facilitated by ligand-functionalized NEs [150] to promote specific cellular uptake and transcytosis; and (c) immunomodulation, which is particularly relevant for vaccine delivery, involving the incorporation of immunostimulatory compounds to enhance immune responses [152]. This can be achieved through adjuvant integration [153] and antigen presentation [154] enhancement. In most cases, developing controlled release systems can optimize drug pharmacokinetics. This includes designing stimuli-responsive formulations [155] and in situ gelling systems that undergo sol–gel transition in the nasal cavity [156] among others.

The mucoadhesive properties of NEs contribute significantly to increased residence time opposing the rapid clearance by the mucociliary system, leading to superior drug absorption, sustained release, and improved bioavailability while minimizing the need for frequent dosing. Mucins—the main components of mucus—are negatively charged, and cationic surfactants can electrostatically interact with them. However, this earlier used approach was not favored due to surfactants’ toxicity. Instead, incorporating mucoadhesive polymers into NEs enhances the mucoadhesive properties of the final formulation either by mucus interaction or even by gel formations upon contact with the nasal mucosa, which is triggered by various stimuli such as changes in temperature, pH, or ionic strength. Poloxamer 407 (Pluronic^®^ F127), a non-ionic synthetic surfactant, is among the most widely studied polymers due to its gelation temperature (~34 °C), which aligns with the internal temperature of the nasal cavity, providing extended residence time. NEs containing poloxamer 407 and hydroxypropyl methylcellulose (HPMC) for olanzapine delivery have demonstrated enhanced retention, resistance to mucociliary clearance, and improved nasal absorption in both ex vivo and, most importantly, in vivo models [157]. Another study examining the in vivo accumulation of Pluronic^®^-based NEs following intranasal administration in the brain revealed the localization of temozolomide of a triacetin/poloxamer derivatives/Transcutol^®^/Labrasol^®^ system, with minimal drug distribution in the GI tract. This underscores the system’s effectiveness and its potential to reduce off-target side effects [158]. The concentration of poloxamer plays a significant role, and requires careful adjustment as increased mucoadhesiveness could lead to the entrapment of the NE-encapsulated drug in the mucus. A study on glioblastoma treatment indicated that increasing the concentration of poloxamer 407 from 5% to 12.5% enhanced the mucoadhesive properties of the formulation, improving topical application. However, it reduced the drug’s penetration through porcine mucosa, with 10% poloxamer being the optimal concentration for mucoadhesive NEs and efficient drug delivery, leading to a 40% reduction in brain tumor size in rats with ~220 nm NEs. Interestingly, Cryo-TEM studies suggested a micelle-like structure around the NE while showing no signs of toxicity in the nasal mucosa and reduced systemic presence [155]. Other systems have proven no ciliotoxicity of NEs with poloxamer 407 [159]. Beyond poloxamers, other synthetic polymers such as PEG and Carbopol^®^ are also used in NE functionalization for intranasal delivery. PEGylation can reduce particle aggregation due to steric repulsion in mucus and can improve particle diffusion. PEG-400 is frequently used, especially in combination with Tweens^®^, while modified PEGs like PEG-660-stearate [159] or vitamin E succinate-PEG 1000 are employed to enhance carrier permeability through their brush-like structure (~3 nm extended), aiding penetration into the nasal mucosa. A detailed structural study using small-angle X-ray scattering (SAXS) of PEG-12-15 hydroxystearate (Solutol^®^ HS 15) NEs demonstrated that PEG prevents entrapment in mucus, facilitating efficient transmucosal delivery due to its mucus-modulating and mucopenetrating properties [150]. The molecular weight of PEG plays a fundamental role in its mucopenetrating properties, with higher molecular weights (>10 kDa), leading to interaction with mucins, while molecular weights of 2–6 kDa are ideal for optimal performance, alongside density considerations [160]. Acrylic polymers, with most representative Carbopols^®^ exert mucoadhesive properties at low concentrations and have been applied to slow drug releasedue to its network [161]. For convulsion treatment gabapentin-loaded mucoadhesive NE with Carbopol^®^ at a concentration of 0.5–0.7% in the total negatively charged NE composed of Tween^®^ 80, PEG-400 and Capmul^®^ MCM increased the residence time of the drug in the mucosa [162]. Patel and colleagues developed a six-month stable lurasidone mucoadhesive NE for the management of schizophrenia, which is one of the most stable NEs described in the literature, making this nanoformulation more appropriate for clinical practice. Their study identified that the system encapsulating 0.5% *w*/*w* of Noveon^®^ AA-1 Polycarbophil was non-toxic and non-irritating to sheep nasal mucosa [163]. Apart the commonly used agents, proteins such as concavalinA have also been grafted into nasal NEs for mucoadhesive properties for specific binding to the mannose and glucose residues of the mucosal glycocalyx [164].

Natural polymers are usually added in NEs due to their low toxicity profile. Chitosan refers to a big category of compounds of different molecular weights, degrees of deacetylation and different modifications, including N-trimethyl chitosan, thiolated chitosan, carboxymethyl chitosan, and others. Chitosan is added in the final formulation, usually dropwise from an acetic stock solution in concentrations ranging from 0.1% [165] to 1% *w*/*v* [166]. An amount of 0.1% of chitosan was used in a cationic (+20 mV) lecithin/MCT/Tween^®^ 80 system of 250 nm size for the delivery of temozolomide for melanoma metastasis brain-related cancer. The in vitro formulation increased the toxic effect of the drug in melanoma cells, implicating the increased cellular uptake, which was also reported previously [167], while no histological alterations following intranasal administration were reported [165]. The systems led to a 71.6% reduced tumor size in xenografic mice with a higher brain distribution even 1.5 h post-administration [168]. Diedrich et al. [167] developed a luteolin NE in the presence of chitosan for effective brain targeting after intranasal administration in a child suffering from neuroblastoma. The formulation exhibited 85.5% encapsulation efficiency and prolonged in vitro release of luteolin up to 72 h. In the in vivo intranasal delivery of lipophilic BNN27, for the treatment of CNS disorders, showed a 3.5-fold increase in brain drug deposition in mice after 1 h when using a 0.3% chitosan, compared to non-chitosan-containing systems [169]. It must be pointed out that the term “chitosan-coated NEs” could be misleading, as it implies an outer layer of chitosan around the NE’s surface; however, the literature lacks supporting structural evidence. Studies using electron paramagnetic resonance (EPR) spectroscopy have proven interactions between chitosan molecules and surfactant membrane in microemulsions [170], pointing out that interactions should be studied on a case-by-case basis due to the variation in components. Other natural components have been used, such as hyaluronic acid, xanthan, alginate, and pectin [171]; however, in recent years, their use seems to have been limited. The rationale behind preparing chitosan NEs, beyond its mucoadhesive nature, lies in its barrier-penetrating capabilities by transiently opening tight junctions [170] between epithelial cells, facilitating drug absorption. Furthermore, is easily modifiable [172], enhancing its applicability for specific drug delivery needs. Its biodegradability, safety, and approval for pharmaceutical applications make it a preferred choice in drug delivery systems, including nasal NEs [173]. Poloxamers, on the other hand, serve dual purposes in formulation: they act as surfactants and provide thermo-responsive properties [155], enabling the development of in situ gels [158]. Their cost-effectiveness and thermosensitive behavior further contribute to their widespread use in nasal NEs. The reduced use of other polymers, such as hyaluronic acid, alginate, and xanthan, may be attributed to several factors. While they possess mucoadhesive properties, they generally lack the permeation-enhancing effects of chitosan or the thermosensitive behavior of poloxamers. Their gel-forming properties can also present challenges in optimizing formulations for nasal delivery, further limiting their adoption. It is also worth noting that hyaluronic acid has been used more for its hydrating and lubricating properties [174]. The combination of mucoadhesive compounds such as poloxamer 407 and gellan gum has also been studied. An in situ nanoemulgel of 106 nm, albeit with an increased PDI, was developed with the use of the ion-sensitive (gellan gum) and the temperature-sensitive (poloxamer 407). The administration of amisulpride (for schizophrenia) in high concentrations (5 mg/kg) in the absence of mucoadhesive compounds led to mortality (two of the six animals died), while the gelling agents prolonged the residence time of the formulation in the nasal cavity with limited permeation of the drug into the blood, with brain targeting and no reported deaths [175]. Along the same lines, semi-synthetic polymers such as cellulose derivatives, mainly hydroxypropyl methyl cellulose (HPMC) and carboxymethyl cellulose (CMC), are included in NEs intended for nasal administration. HPMC increases their residence in the nasal cavity mainly through non-specific interactions at a concentration of 0.3% [176].

Active targeting, in contrast to the previously mentioned passive strategy based on non-specific interactions, involves modifying the surface of NEs with ligands. This approach includes receptor targeting nasal epithelial cells or neurons using proteins such as transferrin (Tf), lactoferrin, or lectins, as well as peptides. Receptor-mediated targeting specifically refers to the modification of NE droplets with ligands that bind to receptors overexpressed on target cells or tissues. Tf is a glycoprotein responsible for transferring iron and has been recently used in NEs functionalization, targeting the CNS for the alleviation of HIV-associated neurocognitive disorder. The method used involves a commonly employed conjugation technique for phospholipids, utilizing the reaction between the maleimide groups of DSPE-PEG2000-maleimide and the thiol groups of the protein. The Tf-functionalized 130 nm O/W NE, composed of 1,2-distearoyl-sn-glycero-3-phosphocholine(DSPC)/DSPE-PEG2000/cholesterol/docosahexaenoic acid, hosted the antiretroviral agent darunavir, which is not brain-selective. Tf receptors are amongst the most studied and have been shown to be promising molecular probes for targeted drug delivery to the brain. Since the BBB prevents the free diffusion of iron into the brain tissue, the brain needs a specialized system that facilitates such uptake and Tf receptors are present on the endothelial cells of the brain but not on endothelial cells elsewhere in the body, which makes them very interesting targets. However, although the system has not been used for nasal delivery—only intravenously—it is a promising start for targeting the brain through functionalization of NEs [177]. Lactoferrin (Lf) an iron-binding cationic glycoprotein (MW 80 kDa) of the Tf family, which plays a physiological defense role against infections and severe inflammation, was observed to lead to increased drug concentrations in rat brains compared to both drug solutions and non-modified NEs. In this case, Lf was added dropwise to the NE systems of IPM, Cremophor^®^ EL, Labrasol^®^, and Capryol^®^90 without any modification [178,179]. The EDC/NHS (1-ethyl-3-(3-dimethylaminopropyl)carbodiimide/*N*-hydroxysuccinimide) [180] strategy also proved applicable in the Lf-modification of NEs, which led to the increased residence time of the indinavir drug in the brain. 

Apart from organ targeting, immunization is another area of interest with cancer immunotherapy to gain significant attention in recent years. Immunization, whether through vaccines or other immune-stimulating agents, can potentially enhance the body’s natural ability to recognize and fight cancer cells. Yang et al. developed a novel NE vaccine (epitope–peptide vaccine) combining the integrin-targeting peptide IKVAV with the tumor antigen epitope OVA 257–264 (I-OVA) to enhance nasal immunization for tumor therapy. Epitope peptides have low immunogenicity and nasal permeability but their attachment in integrin targeting peptides could help to achieve higher antigen uptake through epithelial cells which express different integrins. This O/W NE of an approximate size of 23 nm, was produced by the low-energy emulsification method, and was composed of squalene (SQ) as the oil phase and Tween^®^ 80. It was able to pass through nasal mucosa and migrate to the lymph, resulting in a significantly reduced tumor volume compared to the peptide alone [181]. Epitope peptides modified NEs were also used against *Helicobacter pylori* infection. HpaA epitope peptide (protein/antigen found in this bacteria) in conjunction with a vaccine adjuvant decreased bacterial colonization in *H. pylori*-infected mice with the intranasal administration of IPM as the oil phase, EL-35^®^ as the surfactant, and propylene glycol as the co-surfactant. In this case, the epitope was not conjugated in a NE component but added dropwise in the formulation, which was presumably (without structural evidence) located on the interface of the system, as the size and z-potential revealed. The use of the NE increases the immune response eliciting from the epitope peptide with a more robust immune response [154].

**Figure 5:**
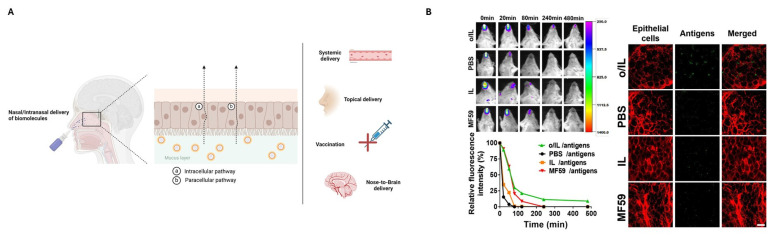
(**A**) Schematic representation of the routes of intranasal drug delivery and its application. Created in BioRender. Mitsou, E. (2025) https://BioRender.com/n01l841, accessed on 1 January 2025. (**B**) Oil-in-ionic liquid NE-based intranasal delivery system for influenza split-virus vaccine and the determination of antigen deposition and permeation in the nasal cavity. In vivo fluorescent images and the semiquantitative analysis of the relative fluorescence intensity of Cy7-SE-labeled antigens in the nasal cavities of immunized mice at different time intervals after the intranasal administration of antigens in different formulations. Confocal laser scanning microscopy images of nasal epithelia at 1 h post-intranasal administration of Cy5-labeled antigens formulated in different vaccine delivery systems. Obtained with permission from [183].

Nasal mucosa is also partially responsible for identifying microorganisms through the recognition of specific pathogen-associated molecular patterns, including Toll-like receptors (TLRs). Experimental mucosal adjuvants containing TLR agonists can stimulate innate immune responses and promote protection against pathogen challenge. A simple cetylpyridinium chloride (CPC)/Tween^®^ 80/ethanol/soybean oil NE not only enhanced antigen uptake but also enhanced uptake in ciliated epithelial cells, without local inflammation as the conventional adjuvants [152]. The same NE with HSV-2 gD2 and gB2 (20 μg of each antigen per dose) surface glycoproteins as antigens showed this vaccine to be a successful prophylactic and therapeutic tool against the herpes simplex virus (HSV) in in vivo studies in guinea pig models [182]. Apart from the conventional O/W NEs, to enhance vaccination efficacy, alternative systems of oil-in-ionic liquid (O/IL) have been developed. Ionic liquids (ILs), which are organic/inorganic salts with melting points below 100 °C, improve the penetration of biomacromolecules through mucosal barriers and produce a strong immune response, while from a scale-up point of view, they can improve the thermostability of the product. One such O/IL NE was developed, composed of choline and niacin IL ([Cho][Nic]), SQ, and Tween^®^ 80 as surfactants with particle size of 168 nm. When used with influenza split virus as an antigen, this NE elicited strong mucosal and systemic immune responses. In studies with H1N1 antigens, intranasal immunization with the O/IL NE resulted in a secretory immunoglobulin titer 25-fold higher than plain antigens and 5.8-fold higher than those with commercial adjuvants, most importantly without signs of toxicity. This approach highlights the potential of O/IL NEs for effective and safe intranasal vaccine delivery (Figure 5) [183].

Overall, the use of NEs for intranasal applications is a highly promising approach, ranging from conventional drug delivery to gene delivery and vaccination. Particularly for vaccination purposes, it has garnered interest, with clinical trials having already completed Phase 1 (Table 2) and shown promising results.

### 3.5. Intra-Articular Administration

Joint diseases affect millions of people worldwide, with rheumatoid arthritis and osteoarthritis being the most prevalent among them, typically involving the knees and the hips. Stiffness, reduced mobility, swelling, and pain are common symptoms, impacting individuals’ well-being and underscoring the urgency for effective treatments. To avoid the costly, painful, and high-risk surgical interventions for alleviating the symptoms of joint diseases, effective drug delivery strategies (i.e., liposomes, lipid and polymeric nanoparticles, hydrogels, etc.) have been developed. So far, as no cure has been found for these multifactorial diseases, the development of efficient drug delivery systems or regenerative approaches is of high importance. While systemic administration has been commonly used in the past decades, the topical manifestations of joint diseases require high drug concentrations, with challenging targeting and a high risk of side effects. For instance, NSAIDs administered systemically for osteoarthritis can cause significant adverse effects in the GI tract and renal system, restricting their use in high-risk patients. As a result, intra-articular (IA) administration—the direct injection of the drug into the joint area—is regarded as a strategy to reduce off-target side effects, minimize required drug concentrations, and achieve high local concentrations.

To offer extended retention time, better drug solubility, sustained release, and targeting (i.e., articular cartilage, chondrocytes, macrophages, etc.) nanosystems such as liposomes, nanoparticles, NEs, dendrimers, hydrogels, and more sophisticated combinations of these have been employed. Although NEs have been utilized in the transdermal drug delivery for joint treatment, their use in IA administration remains limited. NEs for IA delivery of drugs are mainly composed of surfactants such as Tweens^®^ and Spans^®^. The sizes are in the range of 70–250 nm which can lead to different retention profiles. One of the reasons for IA administered drugs encapsulated in NEs is the reduction in side effects caused by the drugs. For example, colchicine for gout arthritis is a potent drug, causing neutropenia, nausea, and bone marrow problems when orally administered. An O/W NE composed of IPM/Span^®^ 60/ethanol was used for the encapsulation of colchicine, with a size of ~103 nm and PDI of 0.2. Upon IA injection, the pharmacokinetics of the radiolabeled drug exhibited lower renal accumulation and lower blood presence with significantly increased retention time in the inflamed joint in a mice model [184]. For targeting the cartilage, nanosystems are typically modified with peptides (e.g., WYRGRL or TAT) or other compounds, although passive targeting via a surface positive charge is also commonly used. Cassic acid-loaded NEs were developed, as cassic acid has properties that may halt osteoarthritis progression. The drug was bound to octadecylamine for a strong positive charge, while chondroitin sulfate (CS), a naturally occurring glycosaminoglycan and an important structural component of cartilage, was used for its targeting properties due to its interactions with collagen type II and chondrocyte receptors. NEs comprising Compritol^®^ 888 ATO (a versatile excipient commonly used for controlled release applications and as a lubricant in solid oral dosage forms), Pluronic^®^ F-127, and cassic acid–octadecylamine were prepared via melt–emulsification–ultrasonication. The NE without CS had a zeta potential of 42 mV, while the one with CS coating had a zeta potential of around −28 mV. Notably, this study used both non-osteoarthritic and osteoarthritic rats, an experimental design that is uncommon, yet extremely useful as it can provide valuable insights into the different behaviors of healthy and diseased animals. In both healthy and arthritic groups, NEs showed the strongest fluorescence signals, with the ability to reach chondrocytes in the osteoarthritis model and sustain fluorescence up to 21 days. Additionally, NEs with CS but without cassic acid reduced the OARSI score compared to the untreated osteoarthritis group, suggesting that other system properties, such as lubrication or antioxidant/anti-inflammatory properties, may benefit the joints. Overall, both passive and active targeting were effective in this study, though active targeting demonstrated a more prolonged effect [185]. Lecithin, zein, eugenol, and GelucireR^®^ 44/14 were used to develop a NE for encapsulating atorvastatin calcium, a chondroprotective agent. Hyaluronic acid was also included in the formulation and poloxamer (15% *w*/*v*) was employed for its thermos-responsive properties. The system was IA-administered in an arthritis model at a dose of 0.4 mg/mL/kg once a week for two weeks. The system displayed a biphasic drug release profile, with a gelation time of 57 s and good syringeability. After 144 h, 50% of the drug was released from the NE thermogel, compared to 90% release from its non-hydrogel counterpart, indicating prolonged release. In vivo, the system reduced swelling in arthritic joints on days 21 and 28 and demonstrated the downregulation of inflammatory markers, including IL-1β, IL-6, and TNF-α, with improvements in overall joint histology [186].

**Figure 6:**
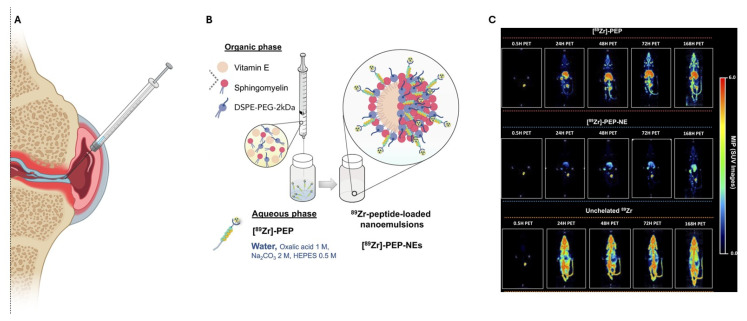
(**A**) Schematic representation of IA injection. Created in BioRender. Mitsou, E. (2025) https://BioRender.com/p79s868, accessed on 1 January 2025. (**B**) O/W NEs composed of vitamin E, sphingomyelin, and a lipid PEG, functionalized with [^89^Zr]-senolytic peptide (PEP). (**C**) Whole-body positron emission tomography (PET) imaging tracer distribution after IA injection of [^89^Zr]-PEP solution, [^89^Zr]-PEP NE, and unchelated ^89^Zr. Obtained with permission from [188].

In addition to their targeting properties, peptides can act also as drugs but, most importantly, can be used as platforms for modifying NEs into theranostic systems. A senolytic peptide (PEP; eliminates or suppresses senescent cells in joints) has been used for the active targeting of chondrocytes and as a new strategy for imaging purposes. The nuclear imaging technique, positron emission tomography (PET), was used for the highly sensitive monitoring of the NE system’s biodistribution, demonstrating its potential as a theranostic tool. The NE system, prepared using an ethanolic injection method, consisted of vitamin E, sphingomyelin, and a PEG lipid, with a particle size of 145 nm and a positive charge of +19 mV. The system remained stable in terms of size in both human plasma and synovial fluid over a one-week period. The peptide was attached through a maleimide-cysteine linkage with C18-PEG6, with radiolabeling occurring at the end of the peptide to ensure exposure. In this study, normal rats received IA injections of the modified NEs, which showed that zirconium-89 (⁸⁹Zr)-PEP was quickly distributed throughout the body, while [⁸⁹Zr]-PEP-NE was retained at the injection site for longer, with reduced accumulation in the liver and kidneys due to prolonged joint retention. Although the primary purpose of this study was to examine biodistribution, the technique holds significant promise for the early detection of joint diseases before severe symptoms appear by employing peptide radiolabeling with the isotope ⁸⁹Zr (Figure 6). This research lays the foundation for developing imaging NEs for osteoarthritis and other joint diseases [187]. 

NEs have recently also been used for cartilage regeneration, a field receiving significant attention by researchers. Sulforaphane (SFN), a compound of anti-inflammatory and antioxidant properties, was recently incorporated into a NE by Selehamin et al. and subsequently embedded within a chitosan/gelatin/PEG scaffold hydrogel for tissue regeneration. Tannic acid (TA) was used as a crosslinker to enhance adhesion to the implantation site while maintaining the hydrogel’s integrity under high-load conditions. SFN served as the oil phase in a simple Tween^®^ 80 NE with a particle size of 75 nm and a slightly negative charge, which was then incorporated into the hydrogel by straightforward mixing. In an in vivo rat model, cartilage defects were created. TA was first applied on the site of defects followed by hydrogel implantation, and an additional TA administration was performed at the defect site. However, instead of the typical IA administration method using a syringe, the developed system was applied directly to the cartilage after a surgical incision. NE decreased the modulus of the hydrogel, but its elongation increased, providing enhanced elasticity and cytocompatibility with chondrocytes. Four weeks post-implantation, histological staining revealed a smoother and more collagen-rich surface in the treated defects, with an improved proteoglycan-to-hyaluronic acid ratio and elevated collagen II production [188].

Overall, NEs in IA delivery have shown promise in overcoming the limitations of traditional drug delivery methods, potentially leading to more effective treatments for conditions such as osteoarthritis and rheumatoid arthritis. Sizes between 100 and 200 nm are commonly employed, while active targeting is of great importance for increased interaction with specific areas within the joint. Positive charge benefits from interactions with collagen II, the main component of articular cartilage, while gelation compounds contribute to sustained release. IA-administered NEs are applications of great interest; however, extensive toxicity profile studies are required as only cytotoxicity studies have been reported in the literature.

### 3.6. Other Routes of Administration

In addition to the routes described above, NEs have been explored in several other drug administration routes, including pulmonary [189], vaginal [190], rectal [191], buccal [192], intratracheal [193], and others. Among these routes, the pulmonary has been the most extensively studied with the co-administration of drugs [194] and other interesting approaches such as the use of deep eutectic solvents [195]; however, in recent years, interest has shifted towards the development of appropriate devices for NEs rather than focusing on the structure of the systems [196]. While this review does not cover all potential routes of NE administration, it focuses on those that have seen the most advancement in the past five years.

## 4. Discussion and Future Perspectives

NEs have emerged as versatile and efficient drug carriers, demonstrating significant potential in overcoming biological barriers and enhancing drug delivery through different approaches (surface charge changes, peptide and antibody functionalization, mucoadhesive and mucopenetrating properties, etc.). Their ability to improve drug solubility, stability, and bioavailability, while offering targeted and controlled release has led to their progress from pre-clinical and clinical studies to commercial applications (Table 1 and Table 2). 

Despite these advancements, limitations remain for NEs and must be addressed. The preparation of NEs requires external energy input (i.e., high-pressure homogenization or ultrasonication), which can hinder the cost- and energy-effectiveness of the final system. In parallel, as non-thermodynamically stable formulations, they pose difficulties for large-scale production due to separation, flocculation, or droplet coalescence, further complicating shelf-life extension and reducing their clinical feasibility. Additionally, many formulations incorporate co-surfactants, such as ethanol, to enhance the stability and solubilization of active ingredients, but these raise several concerns such as evaporation during production or storage, leading to undesirable changes in droplet size and stability issues. Limited toxicological assessment and long-term safety data also pose hurdles in clinical translation along with novel synthesized components which need approval from regulatory authorities. Multi-dose regimens, commonly required for the discussed drug delivery routes, have not been extensively studied, which may lead to unexpected toxic effects over time. This makes it critical to conduct thorough in vivo studies in order to evaluate not only acute toxicity but also potential chronic effects, especially when novel formulations such as NEs are used. 

Regardless the limitations, the future of NEs in drug delivery is highly promising. The development of novel surfactants (such as biosurfactants, gemini, and bicephalous) [197] will likely expand the range of applications across various routes while incorporating stimuli-responsive components and functionalizing NEs surfaces could lead to more efficient barrier penetration. NEs are increasingly being explored for the delivery of non-conventional cargoes such as nucleic acids (mRNA, siRNA), holding significant promise for gene delivery. The incorporation of photoacoustic molecules [198] will also become more widespread for theranostic purposes. Additionally, the growing interest in combining NEs with other nanocarriers (e.g., liposomes, hydrogels [199], and 3D–printed tablets [200]) may further optimize delivery strategies. Cell-camouflaged NEs have garnered significant attention as they are one of the most efficient ways to deliver drugs and penetrate biological barriers by leveraging cell membrane properties [201].

In conclusion, while limitations are still present, ongoing innovations will undoubtedly broaden the application possibilities of these nanocarriers, contributing further to the development of more reliable and effective therapeutic options. 

## Figures and Tables

**Table 1:** Marketed NE-based products.

Marketed Nanoemulsions					
Product Name	Administration Route	Purpose	Information	Licensing Authority	Year and Company
Intralipid^®^/ Vitalipid^®^	Intravenous	Parenteral nutrition for adult and pediatric patients	Soybean oil, egg phospholipids, peanut oil, glycerin, fat soluble vitamins	Swedish Licensing Authority/FDA	1962 Fresenius Kabi (Bad Homburg, Germany)
Cleviprex^®^	Intravenous	High blood pressure	Clevidipine butyrate O/W NE, soybean oil, glycerin, purified egg yolk phospholipids, oleic acid, disodium edetate, and sodium hydroxide	FDA EMA	2008 2021 Novartis (Basel, Switzerland)
Restasis^®^	Ocular	Chronic dry eye disease	Cyclosporine (0.05%) O/W NE, glycerin, castor oil, polysorbate 80, carbomer copolymer type A, purified water, and sodium hydroxide	FDA	2003 Allergan (Dublin, Ireland)/ AbbVie (North Chicago, Illinois, USA)
Ikervis	Ocular	Severe keratitis in dry eye disease patients	Cyclosporine (0.1%) O/W NE, medium-chain triglycerides, cetalkonium chloride, glycerol, tyloxapol, poloxamer 188, sodium hydroxide, water	EMA	2015 Santen Pharmaceuticals (Osaka, Japan)
Durezol^®^	Ocular	Pain and inflammation associated with ocular surgery	difluprednate (0.05%) OW NE, boric acid, castor oil, edetate disodium, glycerin, polysorbate 80, sodium acetate, sodium hydroxide, water for injection	FDA	2008 Sirion Therapeutics (Tampa USA)/Alcon (Geneva Switzerland)
Xelpro^®^	Ocular	Reduction in elevated intraocular pressure in patients with open-angle glaucoma or ocular hypertension	Latanoprost (0.005%) O/W NE, castor oil, polysorbate 80, tyloxapol, glycerin, sodium chloride, carbomer 974P, edetate disodium, sodium hydroxide/hydrochloric acid, water	FDA	2018 Sun Pharmaceutical Industries (Mumbai, India)
Systane^®^ Complete	Ocular	Lubrication and tear film stabilization	Propylene glycol, hydroxypropyl guar, mineral oil, dimyristoyl phosphatidylglycerol, polyoxyl 40 stearate, sorbitan tristearat, boric acid, sorbitol, edetate disodium, POLYQUAD (preservative), water	FDA EMA	2018 Alcon (Geneva, Switzerland)
Cationorm^®^	Ocular	Hydration for sensitive eyes, cataract surgery aftercare	Mineral oils, glycerol, tyloxapol, poloxamer 188, tris hydrochloride, tromethamine, cetalkonium chloride water	EMA	2008 Santen Pharmaceuticals (Osaka, Japan)
NBF Gingival Gel^®^	Oral/Topical	Treatment of oral wound	Aloe vera, ascorbic acid, calcium glycerophosphate, cellulose gum, eucalyptus oil, grapefruit extract, green tea extract, hydrated silica, PEG-32, peppermint oil, pot marigold extract, propolis extract, sorbitol, steviol glycosides, tocopheryl acetate, water, Xylitol	FDA	2017 NanoCureTech Co. Ltd. (Seoul, South Korea)
Ameluz^®^	Dermal	Photodynamic therapy for actinic keratosis	Aminolevulinic acid hydrochloride (10%) gel with NE, xanthan gum, soybean phosphatidylcholine, polysorbate 80, medium-chain triglycerides, isopropyl alcohol, dibasic sodium phosphate, monobasic sodium phosphate, propylene glycol, sodium benzoate, water	FDA EMA	2016/2024 (increased concentration) Biofrontera AG (Leverkusen, Germany)

**Table 2:** Clinical trials on NEs that are either currently active or were completed between 2019 and 2024.

Clinical Trials					
ClinicalTrial ID	Active Compound	Administration Route	Formulation	Application	Status
NCT06188260 (Phase 2, China) RBR-8mj25hx (N/A, Brazil) ISRCTN10208997 (Phase 4, Chile)	Propylene Glycol 0.6%	Ocular	Systane^®^ Complete	Dry eye syndrome (lubricant)	Completed; Ongoing
NCT05724446 (Phase 3, Spain)	Clobetasol propionate	Ocular	Proprietary O/W NE	Post-cataract inflammation treatment in pediatric patients	Active; Recruiting
NCT04249076 (Phase 3, USA) NCT04246801 (Phase 3, USA)	Clobetasol Propionate	Ocular	Proprietary O/W NE	Inflammation and pain associated with cataract surgery	Completed
NCT03785340 (Phase 3, USA)	Brimonidine Tartrate	Ocular	NE eye drops	Dry eye treatment	Completed
KCT0004516 (Phase 4, Seoul)	Cyclosporine	Ocular	Ikervis^®^	Dry eye disease	Not yet recruiting
DRKS00028696 (Phase 4, Germany)	-	Ocular	Cationorm^®^	Dry eye disease (lubricant)	Completed
NCT03865992 (N/A, USA)	Curcumin	Oral	NE	Reducing joint pain caused by treatment with aromatase inhibitors (breast cancer patients)	Active; Not recruiting
TCTR20231114007 (Phase 1, Thailand)	Cannabidiol	Oral	Nano-emulsified Cannabidiol (CBD-NE)	Healthy volunteers; Regulating sleep	Not yet recruiting
NCT05837416 (Phase 4, Egypt)	Propolis; vitamins C and E	Topical/Oral cavity	NBF Gingival Gel^®^	Gingival pigmentation treatment	Active (Recruiting)
NCT02367547 (Phase ½, Finland)	Aminolevulinic Acid	Topical	Ameluz^®^	Photodynamic therapy for superficially growing basal cell carcinomas	Active, not recruiting
IRCT20240622062209N1 (Phase 3, Iran)	Boswellia extract	Dermal	Extract, polysorbate 20, ethanol, isopropyl myristate, water	Improvement of symptoms of psoriasis	Recruiting
IRCT20240213061001N1 (Phase 3, Iran)	Hydrocortisone	Dermal	Calendula oil NE containing hydrocortisone	Dermatitis following radiotherapy in breast cancer patients	Recruiting
CTRI/2022/10/046756 (Phase 3, India)	Quercetin	Dermal	NE quercetin gel	Wound healing	Completed
IRCT20190210042676N22 (Phase 2, Iran)	Finasteride	Dermal	Nanoemulgel lecithin, vitamin E, chloroform, gelling, cholesterol, PEG 600, water	Androgenetic alopecia	Recruiting
RBR-8kmydzj (N/A, Brazil)	Testosterone	Transdermal	Undisclosed NE	Hypoactive sexual desire	Data analysis completed
IRCT20150902023864N3 (Phase 3, Iran)	Ibuprofen	Dermal	NE gel	Osteoarthritis	Recruiting
NCT05397119 (Phase 1, USA)	rH5 Flu Vaccine	Nasal	rH5 Flu Vaccine with O/W NE as adjuvant squalene, Tween 80, PBS	Flu (H5N1)	Completed
NCT04148118 (Phase 1, USA)	Recombinant protein (rPA)	Nasal	NE adjuvanted recombinant protein (rPA) based on Nanovax^®^	Anthrax vaccine	Completed
